# Activation of the nonneuronal cholinergic cardiac system by hypoxic preconditioning protects isolated adult cardiomyocytes from hypoxia/reoxygenation injury

**DOI:** 10.1152/ajpheart.00211.2024

**Published:** 2024-05-03

**Authors:** Felix Braczko, Sara Romina Fischl, Jörg Reinders, Helmut Raphael Lieder, Petra Kleinbongard

**Affiliations:** ^1^Institute for Pathophysiology, West German Heart and Vascular Center, University Duisburg-Essen, Essen, Germany; ^2^Department of Toxicology, Leibniz Research Centre for Working Environment and Human Factors, Technical University Dortmund, Dortmund, Germany

**Keywords:** acetylcholine, cardioprotection, intracardiac nervous system, isolated adult ventricular rat cardiomyocytes, nonneuronal cholinergic cardiac system

## Abstract

Activation of the vagus nerve mediates cardioprotection and attenuates myocardial ischemia/reperfusion (I/R) injury. In response to vagal activation, acetylcholine (ACh) is released from the intracardiac nervous system (ICNS) and activates intracellular cardioprotective signaling cascades. Recently, however, a nonneuronal cholinergic cardiac system (NNCCS) in cardiomyocytes has been described as an additional source of ACh. To investigate whether the NNCCS mediates cardioprotection in the absence of vagal and ICNS activation, we used a reductionist approach of isolated adult rat ventricular cardiomyocytes without neuronal cells, using hypoxic preconditioning (HPC) as a protective stimulus. Adult rat ventricular cardiomyocytes were isolated, the absence of neuronal cells was confirmed, and HPC was induced by 10/20 min hypoxia/reoxygenation (H/R) before subjection to 30/5 min H/R to simulate I/R injury. Cardiomyocyte viability was assessed by trypan blue staining at baseline and after HPC+H/R or H/R. Intra- and extracellular ACh was quantified using liquid chromatography-coupled mass spectrometry at baseline, after HPC, after hypoxia, and after reoxygenation, respectively. In a subset of experiments, muscarinic and nicotinic ACh receptor (m- and nAChR) antagonists were added during HPC or during H/R. Cardiomyocyte viability at baseline (69 ± 4%) was reduced by H/R (10 ± 3%). With HPC, cardiomyocyte viability was preserved after H/R (25 ± 6%). Intra- and extracellular ACh increased during hypoxia; HPC further increased both intra- and extracellular ACh (from 0.9 ± 0.7 to 1.5 ± 1.0 nmol/mg; from 0.7 ± 0.6 to 1.1 ± 0.7 nmol/mg, respectively). The addition of mAChR and nAChR antagonists during HPC had no impact on HPC’s protection; however, protection was abrogated when antagonists were added during H/R (cardiomyocyte viability after H/R: 23 ± 5%; 13 ± 4%). In conclusion, activation of the NNCCS is involved in cardiomyocyte protection; HPC increases intra- and extracellular ACh during H/R, and m- and nAChRs are causally involved in HPC’s cardiomyocyte protection during H/R. The interplay between upstream ICNS activation and NNCCS activation in myocardial cholinergic metabolism and cardioprotection needs to be investigated in future studies.

**NEW & NOTEWORTHY** The intracardiac nervous system is considered to be involved in ischemic conditioning’s cardioprotection through the release of acetylcholine (ACh). However, we demonstrate that hypoxic preconditioning (HPC) protects from hypoxia/reoxygenation injury and increases intra- and extracellular ACh during hypoxia in isolated adult ventricular rat cardiomyocytes. HPC’s protection involves cardiomyocyte muscarinic and nicotinic ACh receptor activation. Thus, besides the intracardiac nervous system, a nonneuronal cholinergic cardiac system may also be causally involved in cardiomyocyte protection by ischemic conditioning.

## Graphical abstract

**Figure:**
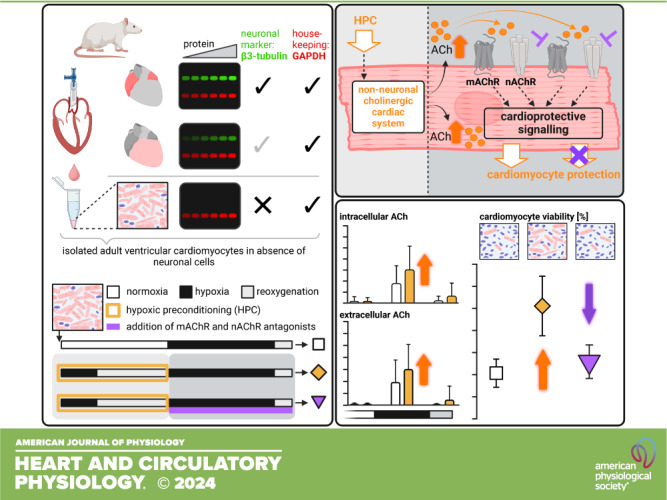

## INTRODUCTION

Vagal activation mediates cardioprotection; activation of efferent vagal nerves reduces heart rate (1) and increases coronary blood flow (2), both of which attenuate myocardial ischemia-reperfusion (I/R) injury and thus reduce myocardial infarct size (3). Activation of efferent vagal nerves reduces infarct size not only when applied during ischemia (4) but also when applied immediately before reperfusion (5, 6). Vagal activation, however, reduces infarct size even in the absence of heart rate reduction in experimental approaches (5–8). Also, in patients with ST-segment elevation myocardial infarction, vagal stimulation through noninvasive low-level tragus stimulation reduced arrhythmias, improved left ventricular function, and attenuated cardiac biomarker release during reperfusion (9).

Cardioprotection by either electrical stimulation or ischemic conditioning (remotely or locally at the heart) (10–12) involves vagal activation and activates the intracardiac nervous system (ICNS), which results in the release of the parasympathetic neurotransmitter acetylcholine (ACh) (13, 14, 18). Parasympathetic postganglionic nerves of the ICNS extend epicardially from the parasympathetic ganglia located in the atrial and ventricular septum to innervate mainly the atria, the interatrial septum, and also the ventricles (15). Vagal innervation of the ventricle is sparse but evident in humans, large mammals, and also in rodents (14–18). In rat and rabbit hearts, local ischemic preconditioning (IPC) was associated with an increase of endogenous, myocardial ACh (14, 19), and cardioprotection was abrogated by the muscarinic AChR (mAChR) antagonist atropine, as well as by the nicotinic AChR (nAChR) antagonist hexamethonium (14). As nAChRs are expressed on ganglia of the ICNS (20), it was suggested that the ICNS is causally involved in IPC’s cardioprotection (14). However, nAChRs, particularly α-7nAChRs (α7nAChR), are also expressed on adult rat cardiomyocytes (21). Several nAChR agonists not only reduce infarct size in rat hearts (22–25) but also prevent cell death after hypoxia/reoxygenation (H/R) of cultured primary neonatal cardiomyocytes (26). Moreover, expression of choline acetyltransferase (ChAT), vesicular ACh transporter (VAChT), high-affinity choline transporter 1 (CHT1), and ACh esterase (AChE), which enable ACh production, storage, transport, secretion, and degradation, has been reported in cardiomyocytes and defined as the nonneuronal cholinergic cardiac system (NNCCS) (27–31). Indeed, there is preliminary evidence indicating that the NNCCS is involved in cardiac ACh metabolism and ACh-derived cardioprotection. Cardiomyocyte-specific overexpression of ChAT, for example, reduced infarct size in isolated perfused mouse hearts with global I/R and improved survival after permanent coronary artery ligation in vivo (32). However, the role of the NNCCS in mediating immediate cardioprotection is unclear (18).

We therefore aimed to investigate whether NNCCS is involved in immediate cardiomyocyte protection by using a reductionist approach of isolated adult rat ventricular cardiomyocytes. First, we excluded the presence of potentially interfering neuronal cells or fibers in our cardiomyocyte preparation by demonstrating the absence of the neuronal marker protein class III β-tubulin (TUBB3) (33). Hypoxic preconditioning (HPC) was induced in these isolated, neuronal cell-free preparations to simulate IPC on the cardiomyocyte level (34). Simulated I/R injury was induced by subjecting cardiomyocytes to H/R, and cardiomyocyte viability was quantified (35). To validate a potential NNCCS activation, intra- and extracellular ACh was quantified via liquid chromatography-coupled mass spectrometry (LC-MS) at baseline, after HPC, after hypoxia, and after H/R in a subset of experiments, respectively. In another subset, atropine and hexamethonium were added to the cardiomyocytes to address the role of cardiomyocyte mAChRs and nAChRs in HPC’s protection, and cardiomyocyte viability was again quantified. To identify the temporal nature of the NNCCS activation, we added the respective receptor antagonists during HPC or during H/R. To assess the involvement of the AChR receptor subtypes, antagonists were added separately and in combination.

## METHODS

### Materials

The authors declare that all data from this exploratory study are available in the article. All procedures involving animals were conducted in accordance with the German laws for animal welfare and the regulations of the local governmental Animal Care and Use Committee of the Landesamt für Natur, Umwelt und Verbraucherschutz Nordrhein-Westfalen, Germany, and are reported in accordance with the Animal Research: Reporting of In Vivo Experiments (ARRIVE) guidelines (36) and conform to the “Position of the American Heart Association on Research Animal Use,” adopted on November 11, 1984. The experimental protocol for isolation and measurement of cardiomyocyte viability was standard (37, 38) and described in detail previously (35, 39–41). Saline buffers used for cardiomyocyte isolation, HPC, and H/R protocols were based on a Tyrode solution. Unless otherwise specified, all chemicals were purchased from Sigma-Aldrich (Deisenhofen, Germany).

### Isolation of Rat Hearts

Adult male and female Lewis rats (200–380 g, 2.0–3.5 mo, Central Animal Laboratory, University of Duisburg-Essen, Essen, Germany) were used. Rats were euthanized by an intraperitoneal injection of sodium pentobarbital (800 mg/kg, Narkoderm, CP-Pharma, Burgdorf, Germany) in compliance with the American Veterinary Medical Association’s Guidelines for the Euthanasia of Animals (42). After disappearance of the withdrawal reflex, hearts were excised, arrested in ice-cold saline (supplemented with unfractionated heparin 250 IU/mL), and weighed. Isolated hearts were used for cardiomyocyte isolation, as well as for the preparation of atrial and ventricular tissue samples.

### Isolation of Adult Ventricular Rat Cardiomyocytes

Isolated rat hearts (*n* = 46, 23 males, 23 females) were mounted on a modified Langendorff apparatus and perfused with isolation buffer consisting of (in mmol/L) 113.0 NaCl, 4.7 KCl, 0.6 KH_2_PO_4_, 0.6 Na_2_HPO_4_, 1.2 MgSO_4_, 12.0 NaHCO_3_, 10.0 KHCO_3_, 10.0 4-(2-hydroxyethyl)-1-piperazineethanesulfonic acid (HEPES), 30.0 taurine, 5.5 glucose, and 10.0 2,3-butanedione monoxime (pH 7.4 at 36.5°C) at a constant flow of 7 mL/min × g heart wt for 3 min. Liberase (250 µg/mL, thermolysin medium research grade, Roche, Mannheim, Germany) and 12.5 µmol/L CaCl_2_ were subsequently added to the perfusion buffer, and hearts were digested for 4.5 min. Atrial and connective tissue were removed and discarded, ventricles were sectioned, and cells were resuspended in isolation buffer containing bovine calf serum [10% (vol/vol) and CaCl_2_ (12.5 µmol/L)]. Cardiomyocytes were isolated and separated from myocardial tissue residues by filtering through a nylon mesh filter (200-µm pore size, Millipore, Billerica, MA). CaCl_2_ was then slowly titrated at 20°C to a final concentration of 1.0 mmol/L through five steps, each accompanied by cardiomyocyte purification through natural sedimentation by gravity with a duration of 10 min, resulting in a purity of cardiomyocytes of approximately 97% (40). Isolated cardiomyocytes were then kept in incubation buffer under normoxic conditions, containing (in mmol/L) 125.0 NaCl, 5.4 KCl, 1.2 NaH_2_PO_4_, 20.0 HEPES, 5.0 taurine, 15.0 glucose, 2.5 creatine, 0.5 MgCl_2_, and 1.0 CaCl_2_, gassed with 100% oxygen (pH 7.4) in protein low-binding microtubes (Eppendorf, Hamburg, Germany). The method for cardiomyocyte isolation was previously described (35, 40).

### Quantification of Cardiomyocyte Viability

Isolated cardiomyocytes were stained with 0.5% trypan blue and 400–800 cells per sample were analyzed in nonoverlapping visual fields using light microscopy at ×40 magnification (Leica DMLB microscope, Leica, Bensheim, Germany) to determine baseline viability. Viability was expressed as the percentage of rod-shaped, unstained isolated cardiomyocytes over the total number of cells. Isolated cardiomyocyte preparations with a viability of <60% at baseline were discarded (35). Aliquots from isolated cardiomyocytes were frozen in liquid nitrogen and stored at −80°C for Western blot analyses.

### Quantification of Neuronal Marker Protein Class III β-Tubulin via Western Blot

To validate the absence of neuronal cells or nerve fibers in our isolated cardiomyocyte preparation, TUBB3 was analyzed using Western blot technique in *n* = 12 representative cardiomyocyte isolations. The microtubule element TUBB3 is exclusively expressed in neuronal cells not only of the central nervous system but also of the peripheral nervous system (33, 43). As positive controls for TUBB3, atrial and ventricular myocardial tissue samples were separated and rapidly cut into small pieces (*n* = 6 rat hearts; 3 males, 3 females). Two aliquots were extracted from each sample, frozen in liquid nitrogen, and stored at −80°C until further use for Western blot analyses. Frozen samples of atrial and ventricular tissue (∼4–30 mg) and isolated cardiomyocytes were homogenized in 100.0 mmol/L tris(hydroxymethyl)aminomethane with 2% sodium dodecyl sulfate (wt/vol; SERVA Electrophoresis, Heidelberg, Germany), heated to 70°C for 5 min, and centrifuged at 16,000 *g* for 10 min. The protein lysate containing supernatants was stored at −80°C in aliquots. Pooled lysates of atria, ventricles, and isolated cardiomyocytes, respectively, were used to determine the combined linear range for TUBB3 and glyceraldehyde-3-phosphate dehydrogenase (GAPDH) as “housekeeping protein” (2–40 µg in steps of 2 µg). Subsequently, the respective protein quantity within the linear range (22 µg) was used for Western blot analysis of all individual samples (atria, ventricles, and isolated cardiomyocytes, respectively). The samples were applied to precast stain-free 12% sodium dodecyl sulfate polyacrylamide electrophoresis gels (Bio-Rad, Hercules, CA). A reference sample (mixture of lysates from atria, ventricles, and isolated cardiomyocytes) was prepared and loaded on all gels. Total protein was quantified by an ultraviolet light-induced fluorescence reaction of protein-tryptophan with tri-halo compounds within the stain-free gels and imaged using the Gel Doc Go system (Bio-Rad). Proteins were transferred to 0.45-µm low-fluorescence polyvinylidene difluoride membranes (Merck Chemicals, Darmstadt, Germany) using the Trans-Blot Turbo transfer system (Bio-Rad). The membranes were imaged (Gel Doc Go system) and dried. After reactivation with 100% methanol, membranes were blocked (1:10, EveryBlot blocking buffer, Bio-Rad) for 5 min at room temperature, rinsed with tris-buffered saline, and cut horizontally. All wash and blocking steps were carried out on an orbital shaker. The method for Western blot analysis has been previously described (44). The membranes were incubated with the respective primary antibodies directed against TUBB3 (rabbit anti-β_3_-tubulin, Cat. No. 5568, clone D71G9, 1:300, Cell Signaling Technology, Danvers, MA) or GAPDH (rabbit anti-GAPDH, Cat. No. 5174, clone D16H11, 1:1,000, Cell Signaling Technology) overnight at 4°C. Membranes were washed 4 × 5 min with tris-buffered saline containing polyoxyethylene-20-sorbitan monolaurate (TBST) before incubation with the secondary antibody (IRDye 680RD goat anti-rabbit, Cat. No. 926–68071, LI-COR Biosciences, Lincoln, NE), 1:5,000 for TUBB3, 1:20,000 for GAPDH, for 1 h at room temperature. Primary antibodies were diluted in TBST containing 5% bovine serum albumin. The secondary antibody was diluted in EveryBlot blocking buffer (1:10), supplemented with 0.02% polyoxyethylene-20-sorbitan monolaurate. The fluorescence signal intensity of TUBB3 and GAPDH was imaged using the LI-COR Biosciences infrared imaging system (Odyssey Fc). Detected signals were quantified with the LI-COR Biosciences Empiria studio software (v. 1.3.0.83). All signal intensities were normalized to the reference sample to ensure comparability between gels/membranes. The fluorescence signal intensity of TUBB3 and GAPDH of the combined linear range were reported as arbitrary units. For individual samples, the fluorescence signal intensity of TUBB3 was normalized to the respective signal intensity of GAPDH.

### Hypoxic Preconditioning in Isolated Cardiomyocytes

#### Hypoxic preconditioning, hypoxia/reoxygenation, and ACh quantification.

Isolated cardiomyocytes were assigned to the respective protocols (please see Supplemental Fig. S1). The HPC protocol was adapted from isolated adult ventricular mouse cardiomyocytes (34). In isolated cardiomyocytes from *n* = 31 isolated hearts (15 males, 16 females) HPC was induced by 10 min of exposure to HPC buffer (incubation buffer without glucose, gassed with N_2_) under hypoxic conditions in an airtight chamber filled with N_2,_ followed by a 20-min reoxygenation with reoxygenation buffer under normoxic conditions [with an osmolality of 250 mOsmol/L and containing (in mmol/L) 88.0 NaCl, 5.4 KCl, 1.2 NaH_2_PO_4_, 20.0 HEPES, 5.0 taurine, 15.0 glucose, 2.5 creatine, 0.5 MgCl_2_, and 1.0 CaCl_2_, gassed with 100% oxygen (pH 7.4)]. For the respective control without HPC, incubation buffer under normoxic condition was added to the isolated cardiomyocytes for 30 min. The cardiomyocytes were then further divided and assigned to H/R and time control (TC). In cardiomyocytes assigned to H/R, hypoxia was induced for 30 min by exposing isolated cardiomyocytes to hypoxic buffer under hypoxic conditions containing (in mmol/L) 119.0 NaCl, 12.0 KCl, 5.0 HEPES, 0.5 MgCl_2_, 0.9 CaCl_2_, and 20.0 sodium lactate (pH 6.5) and sealed with paraffin oil. Isolated cardiomyocytes were kept in solution where they sediment; reoxygenation was induced by the removal of oil and the addition of reoxygenation buffer under normoxic conditions for 5 min. Isolated cardiomyocytes assigned to TC were kept in incubation buffer under normoxic conditions, and the buffer was replaced at the corresponding time points. Viability of isolated cardiomyocytes was determined at 65 min after H/R and after TC, respectively (Supplemental Fig. S1). The protocol for H/R in isolated cardiomyocytes was previously described (35).

In a randomly selected subset of the above experiments, intra- and extracellular ACh was quantified. In aliquots of isolated cardiomyocyte preparations (subgroup of *n* = 10 cardiomyocyte isolations; 5 male rats, 5 female rats), physostigmine (0.1 mmol/L) was added throughout the protocol to inhibit ubiquitous ACh esterases (27). Both isolated cardiomyocytes and their respective supernatants were sampled at time points after HPC (30 min), after hypoxia (60 min), after reoxygenation (65 min), and the respective TC, frozen in liquid nitrogen, and stored at −80°C until further use (see Supplemental Fig. S1). LC-MS was used to quantify intracellular and extracellular (cardiomyocyte supernatants) ACh (Prolytic, Frankfurt am Main, Germany). Pure acetonitrile containing internal standard (d4-ACh, 25 pg) was added to all samples. Samples were centrifuged at 12,000 *g* for 10 min, and the respective supernatant was then used for LC-MS analyses. Analyses were conducted on a QTrap 6500^+^ mass spectrometer (Sciex, Framingham, MA) coupled to a 1260 Infinity II liquid chromatography system (Agilent, Santa Clara, CA). Five microliters of each sample was applied to a 50 × 2.1 mm internal diameter, 3.5-µm Agilent HILIC Plus column (Agilent) with a flow of 600 µL/min at *solvent A* [15% 10 mmol/L ammonium formate in acetonitrile (vol/vol) and 0.02% formic acid] for 2 min, then *solvent B* [50% 10 mmol/L ammonium formate in acetonitrile (vol/vol) and 0.02% formic acid] was added for 3 min, and then *solvent A* again for 7 min. The LC-MS was operated in turbo ion-spray mode with multiple reaction monitoring using the transitions 146.2–86.9 *m/z* (for ACh) and 150.247–91.0 *m/z* (for d4-ACh) for quantification via the Analyst software (v.1.7.2, Sciex). The lower limit of quantification was 2.0 nmol/L. Protein content of isolated cardiomyocytes was quantified (Pierce Detergent Compatible Bradford Assay, Thermo Scientific, Waltham, MA) to normalize ACh to nanomoles per milligram cardiomyocyte protein.

#### Hypoxic preconditioning with AChR antagonists.

To characterize the HPC-mediated activation of the NNCCS and the temporal nature of the NNCCS activation, the mAChR and nAChR antagonists atropine and hexamethonium, or a combination of both, were added in additional isolated cardiomyocyte experiments from *n* = 9 isolations (4 male, 5 female rats). The antagonists were added to the respective buffers during *1*) the first 30 min with the HPC stimulus or without HPC, *2*) the 35 min of H/R protocol, or *3*) throughout the entire protocol (Supplemental Fig. S1). Atropine (0.1 µmol/L) was used as a mAChR antagonist and hexamethonium (1.0 µmol/L) as a nAChR antagonist. The concentrations of the AChR antagonists were adapted from prior studies (13, 14). The effectiveness of both antagonists and their potential cardioprotective properties were assessed in preliminary experiments (for details see Supplemental Fig. S2, *A* and *B*). The lowest effective concentrations of atropine and hexamethonium were chosen for the experiments and did not impact cardiomyocyte viability per se (Supplemental Fig. S2, *A* and *B*).

### Statistics

Investigators performing isolated rat cardiomyocyte experiments and assessing cardiomyocyte viability were blinded with respect to the protocol. The Kolmogorov–Smirnov test was used to test normality for all datasets, and normality was confirmed for Western blot data and data of cardiomyocyte viability. Normality was not confirmed for data of intra- and extracellular ACh. The data are presented as means ± SD. Rats used for tissue and cardiomyocyte isolation were randomized according to sex for each respective isolation and were marked by using different colors for the respective data points, and combined data were reported (45). Linear regression analyses were used for Western blot fluorescence intensities, and correlation coefficients (Pearson’s *R*) were calculated (OriginPro, v.2023b, OriginLab, Northampton, MA). Two-way analysis of variance (ANOVA; sex, protocol) was used to analyze cardiomyocyte viability for HPC and differences between male and female rats. One-way ANOVA for repeated measures was used to analyze the viability of isolated rat cardiomyocytes with AChR antagonists. Datasets for intra- and extracellular ACh were normalized by square root transformation (38, 46) and were analyzed by two-way ANOVA for repeated measures (time point, protocol). Individual mean values of datasets were compared by Fisher’s least significant difference post hoc tests when the ANOVA indicated a significant difference (SigmaStat 3.5, Erkrath, Germany). Differences were considered significant at the level of *P* < 0.05.

## RESULTS

### Neuronal Marker Class III β-Tubulin is Not Present in Preparations of Isolated Cardiomyocytes

There was a linear correlation between protein concentration and fluorescence signal for GAPDH in the pooled protein lysates from atria, ventricles, and isolated cardiomyocytes, respectively (Supplemental Fig. S3, *A–C*). The slope of the linear regression was comparable for atria and ventricle lysates but lower for isolated cardiomyocytes. In atria and ventricle lysates, TUBB3 correlated with increasing protein concentration, while it was lower in ventricle lysates, confirming prior reports (Supplemental Fig. S3, *A–C*) (15, 16). Full, uncut membranes and the respective fluorescence signals are presented in Supplemental Fig. S4, *A* and *B*. In protein lysates of isolated cardiomyocytes, no TUBB3 signal was detectable (Supplemental Fig. S3*C*). Individual protein lysates of atria, ventricle, and isolated cardiomyocytes confirmed that TUBB3 was most pronounced in atria and less in ventricles, while there was no expression in isolated cardiomyocytes (Supplemental Fig. S4, *C* and *D*). There was apparently no difference in TUBB3 expression between female and male myocardial tissue or isolated cardiomyocytes (Supplemental Fig. S4, *C* and *D*).

### Viability of Isolated Cardiomyocytes with Hypoxic Preconditioning and Intra- and Extracellular ACh

**Figure 1.**
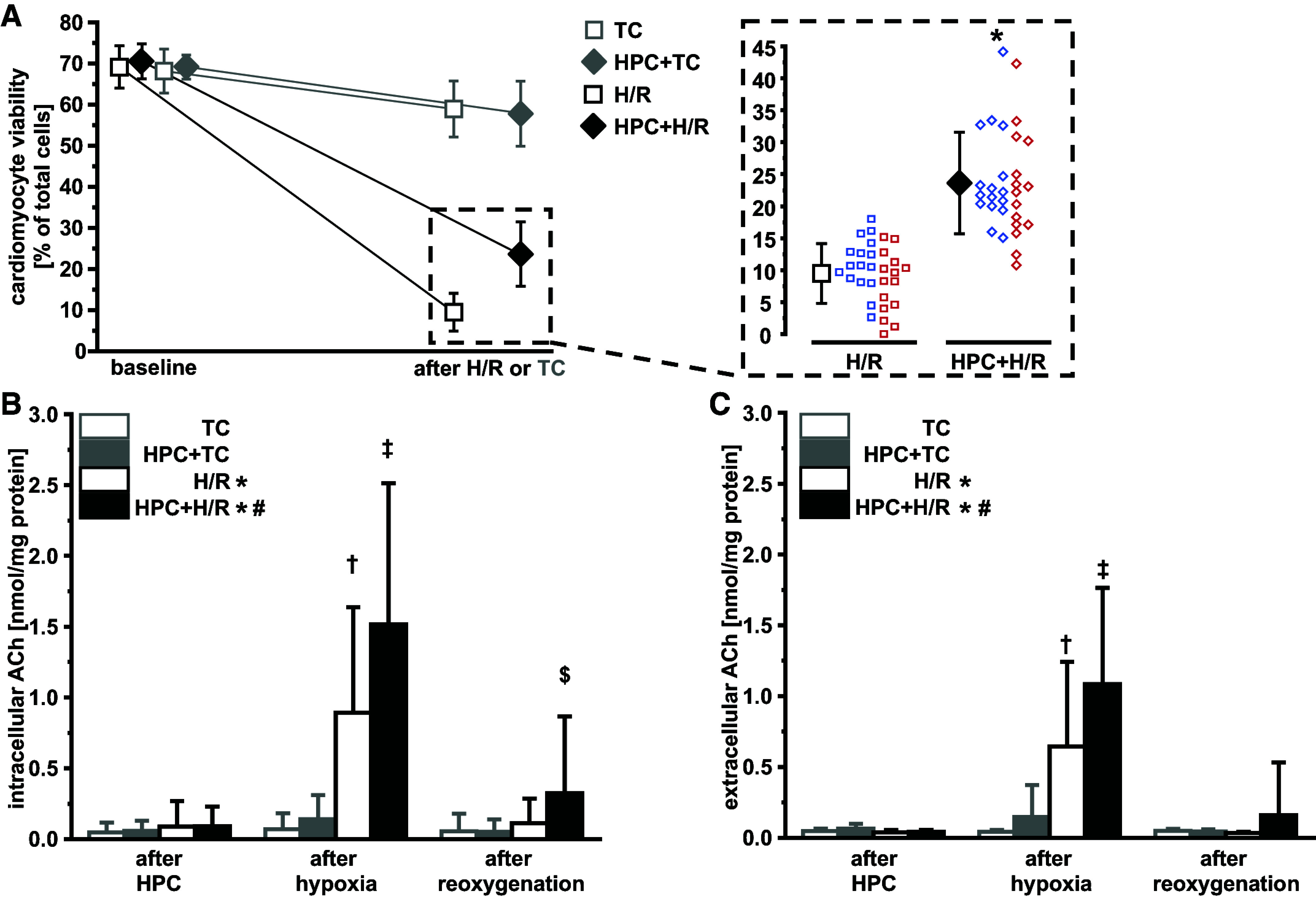
Hypoxic preconditioning preserves viability of isolated adult ventricular rat cardiomyocytes after hypoxia/reoxygenation (H/R; *A*) and increases intracellular (*B*) and extracellular (*C*) cardiomyocyte acetylcholine (ACh). Data are presented as means ± SD. *A*: viability of isolated adult ventricular rat cardiomyocytes at baseline and after H/R (black), or time control (TC; light gray; *left*) and cardiomyocyte viability after H/R (*right*). Cardiomyocytes were isolated from *n* = 31 hearts (16 males, blue data points; 15 females, red data points). Two-way ANOVA (protocol, sex) with Fisher’s least significant differences post hoc tests. *P < 0.001 vs. H/R. *B* and *C*: subgroup of the *n* = 31 (*n* = 10 hearts; 5 males, 5 females). Two-way ANOVA for repeated measures (protocol and time point) with Fisher’s least significant differences post hoc tests of square root-transformed data; between protocols: *P < 0.05 vs. TC and HPC + TC; #*P* < 0.05 vs. H/R, TC and HPC + TC; after hypoxia: †*P* < 0.001 vs. TC and HPC + TC; ‡*P* < 0.01 vs. H/R; after reoxygenation: $*P* < 0.05 vs. all other groups. HPC, hypoxic preconditioning.

**Figure 2.**
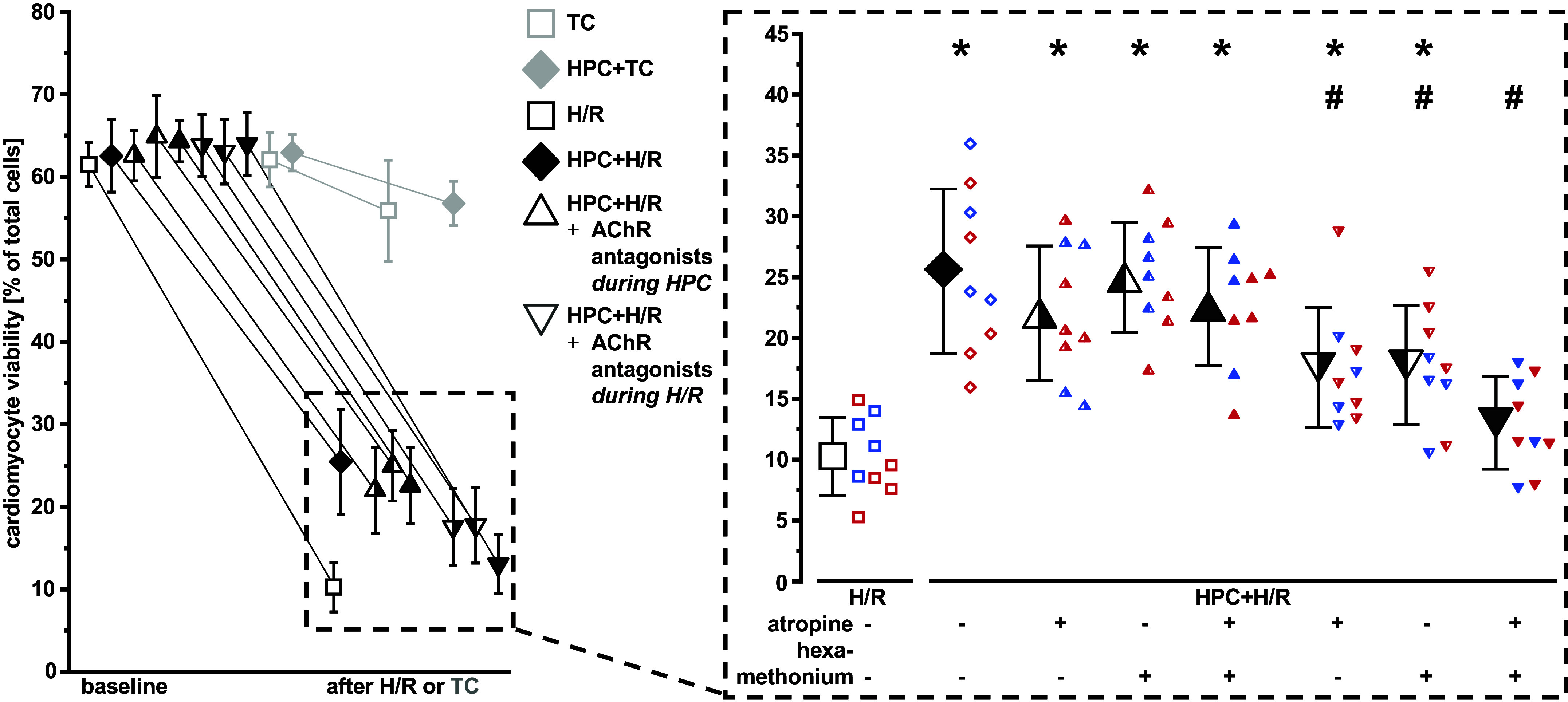
Muscarinic and nicotinic acetylcholine receptor (AChR) antagonists (atropine, hexamethonium, or the addition of both) during hypoxia/reoxygenation (H/R) abrogate cardiomyocyte protection via hypoxic preconditioning (HPC). Data are presented as means ± SD. *Left*: viability of isolated adult ventricular rat cardiomyocyte at baseline and after H/R (black), or time control (TC; gray). *Right*: cardiomyocyte viability after H/R. Half-full triangles on the right indicate addition of atropine, half-full triangles on the left indicate addition of hexamethonium, and full triangles indicate addition of both antagonists. Cardiomyocytes were isolated from *n* = 9 hearts (four males, blue data points; five females, red data points). One-way ANOVA for repeated measures with Fisher’s least significant differences post hoc tests. *P < 0.01 vs. H/R; #*P* < 0.01 vs. HPC + H/R.

Viability of isolated cardiomyocytes ranged between 61% and 73% at baseline, and H/R reduced cardiomyocyte viability to 10 ± 5% (Figs. 1*A* and 2). With HPC, cardiomyocyte viability was preserved to 24 ± 8% (Figs. 1*A* and 2).

Intra- and extracellular ACh at baseline remained mostly below or at the limit of quantification (data not shown). There was an increase of intra- and extracellular ACh during hypoxia. This increase in ACh was more pronounced with HPC (Fig. 1, *B* and *C*), whereas there was almost no increase in ACh immediately after HPC or during the respective time point in cardiomyocytes subjected to normoxic buffer (TC; Fig. 1, *B* and *C*). With HPC, increased intracellular ACh remained elevated during reoxygenation (Fig. 1*B*). Neither antagonism of mAChRs or nAChRs nor a combination during HPC affected HPC’s protection (Fig. 2). Addition of mAChR or nAChR antagonists during H/R, however, attenuated HPC’s protection. The combination of the AChR antagonists during H/R abrogated this protection (Fig. 2). With TC, viability of isolated cardiomyocytes decreased by 6 ± 3% (Figs. 1*A* and 2), regardless of HPC (Figs. 1*A* and 2) or AChR antagonists (Supplemental Fig. S2).

There was no difference in the viability of cardiomyocytes when cells were isolated from male or female rat hearts, nor in their responses to HPC (Figs. 1 and 2).

## DISCUSSION

Here, we extend the concept of the cardioprotective cardiac cholinergic metabolism. We provide evidence that the NNCCS is causally involved in ACh-mediated cardiomyocyte protection. In our reductionist assay of isolated adult rat ventricular cardiomyocytes, where neuronal fibers were absent, HPC increases intra- and extracellular ACh and mediates cardioprotection through m- and nAChR activation. Thus, the ICNS is not the only source of cardiac ACh, as the NNCCS serves as an additional source for cardiac ACh release during hypoxia.

Clearly, ACh is involved in cardioprotection (18, 47–49) and again, the idea that not only the ICNS but also the NNCCS may be involved in cardioprotection by ischemic conditioning is not new (18, 50–52). In the recovery phase after acute myocardial infarction, infarct size reduction by remote ischemic conditioning (RIC) in mice was associated with increased myocardial ChAT and CHT1 expression and with increased myocardial ACh 12–24 h after sustained ischemia (51, 52). However, parasympathetic neuronal fibers/ganglia were verified in the ventricular myocardium of rodents (15, 16, 53), making it impossible to distinguish between neuronal or cardiomyocyte ChAT and CHT1 expression. Thus, the observed cardioprotection could not be attributed to the ICNS or the NNCCS with certainty. Of note, an increase in myocardial ACh or expression of ChAT and CHT1 hours after reperfusion is certainly not causal for acute infarct size reduction (54). Conversely, in studies attributing the cardioprotection to activation of the ICNS (13, 14), the NNCCS’s role in immediate cardioprotection could not be excluded (18).

In experimental studies with an intact ICNS, such as studies in vivo or in isolated perfused hearts, ACh was released into the myocardial interstitium in response to short periods of ischemia, i.e., 5 min of left anterior descending artery occlusion in rabbit hearts (19) and into the coronary effluent after 3 × 5/5 min cycles of I/R in isolated perfused rat hearts (14). ACh increased during short periods of ischemia in rabbit hearts in vivo (19) and after IPC in isolated perfused rat hearts (14). IPC or anesthetic preconditioning with sevoflurane was abrogated through m- or nAChR antagonists in rats in vivo (55) and in isolated perfused rat hearts (14, 56, 57), when applied before (14, 56, 57) or during (55) sustained I/R. In cultured primary neonatal cardiomyocytes (5, 26, 55, 58–60), as well as in isolated adult ventricular cardiomyocytes (61), exogenous ACh (5, 26, 58–61) or a nAChR agonist (55) mediated protection during simulated ischemia. Thus, to exert cardioprotection, the release of ACh and the concomitant activation of m- and nAChRs seems to be relevant during the cardioprotective stimulus as well as during I/R or H/R injury.

In our cardiomyocyte preparation in the absence of neuronal cells, we did not detect ACh release after HPC, but only during H/R. Also, the m- and nAChR antagonists abolished HPC-mediated protection only when added during H/R. ACh was quantified in the presence of the AChE inhibitor physostigmine, thus rendering it impossible to estimate a cardioprotective threshold concentration for ACh; m- and nAChR antagonist-mediated abrogation of HPC’s cardiomyocyte protection indicated, however, a causal relevance of the released ACh.

Thus, it appears that during ischemic conditioning in situ or in vivo, ACh is not released by the NNCCS but rather by the ICNS. However, during sustained ischemia, the respective contribution of ICNS and NNCCS to cardioprotective ACh release cannot be estimated from the present data. Interestingly, the peak extracellular concentration of ACh during hypoxia measured in our reductionist assay of isolated cardiomyocytes, in the presence of the AChE inhibitor physostigmine, is comparable to that measured in the interstitium microdialysate of the area at risk during myocardial ischemia in cats or rabbits (19, 62–64).

Which ACh-independent trigger during ischemic conditioning might be responsible for the subsequent activation of the NNCCS and production of ACh? The autacoids adenosine and bradykinin are known extracellular trigger molecules that are released from cardiomyocytes during IPC (65). They act on sarcolemmal receptors or act receptor-independently and subsequently activate downstream cardioprotective cytosolic signaling cascades, which ultimately converge on subcellular effector structures, such as mitochondria (11, 12, 66). Among the different cytosolic-signaling molecules, the protein kinase C (PKC) may mediate ACh formation during I/R. PKC was described to be causally involved in infarct size reduction by IPC in rabbit hearts (67, 68), and in several cell types (e.g., embryonic kidney or neuroblastoma cells) PKC activated ChAT (69, 70). However, future investigations are needed to clarify this signal cascade(s).

We characterized that m- and nAChRs are causally involved in cardiomyocyte protection via HPC. Although m- and nAChRs are on the outer cardiomyocyte membrane (71, 72), nAChRs are also localized on mitochondria (21, 73). In mouse liver mitochondria, the α7nAChR was involved in the regulation of the mitochondrial permeability transition pore opening (74, 75). The opening of the mitochondrial permeability transition pore is recognized as the final step of the I/R injury, responsible for mitochondrial damage and cardiomyocyte death (66, 76).

### Limitations

We used atropine for mAChR and hexamethonium for nAChR antagonism to better compare our results with previous studies (14) and therefore did not further distinguish between different types of mAChR or nAChR. The present and all previous experimental studies on the NNCCS have been conducted in rodents or rodent cardiomyocytes/hearts (27, 32, 50–52). Thus, whether the NNCCS is also relevant for cardioprotection in other species, especially humans, remains unclear. In human cardiomyocytes, ChAT and VAChT have been identified (77) and nonneuronal cholinergic systems in other human cells/organs support its general presence and relevance in human health and disease (78, 79). Again, the parasympathetic ventricular innervation is more pronounced in larger species (pig, dog, sheep, and human) than in smaller species (mouse and rat) (16). We focused on the relevance of the parasympathetic mediator ACh; however, there is obviously a cross talk between the parasympathetic and sympathetic systems during myocardial infarction (80) and in cardioprotection (81). We used HPC to simulate local ischemic conditioning and focused on its relevance for NNCCS activation. Whether RIC, with its systemic neurohumoral signaling and the spleen as a relay organ in animal models (82) in humans (83), activates the NNCCS remains unclear. It is only described that the humoral signaling involves the ICNS (13), however, with all the limitations of the experimental models used so far.

We focused on cardiomyocytes, which, however, only account for 25 to 35% of the cell composition of a mammalian heart (84, 85). A nonneuronal cholinergic system was also described in leukocytes (78, 79) and endothelial cells (86–88). The nonneuronal cholinergic systems of the different cell types within the myocardium may interact in vivo during cardioprotective ACh signaling.

## DATA AVAILABILITY

Data will be made available upon reasonable request.

## SUPPLEMENTAL DATA

10.6084/m9.figshare.25526173.v3 Supplemental Figs. S1–S4: https://doi.org/10.6084/m9.figshare.25526173.v3.

## GRANTS

This article is based upon the work of the European Cooperation in Science and Technology (COST) Action EU-METAHEART, supported by COST Grant CA22169 (to P.K.).

## DISCLOSURES

P. Kleinbongard is an editor of *American Journal of Physiology-Heart and Circulatory Physiology* and was not involved and did not have access to information regarding the peer-review process or final disposition of this article. An alternate editor oversaw the peer-review and decision-making process for this article. None of the other authors has any conflicts of interest, financial or otherwise, to disclose.

## AUTHOR CONTRIBUTIONS

H.R.L. and P.K. conceived and designed research; F.B. and S.R.F. performed experiments; F.B., S.R.F., and J.R. analyzed data; F.B., J.R., H.R.L., and P.K. interpreted results of experiments; F.B. prepared figures; F.B. and P.K. drafted manuscript; F.B., H.R.L., and P.K. edited and revised manuscript; F.B., S.R.F., J.R., H.R.L., and P.K. approved final version of manuscript.
